# Friends with social benefits: host-microbe interactions as a driver of brain evolution and development?

**DOI:** 10.3389/fcimb.2014.00147

**Published:** 2014-10-29

**Authors:** Roman M. Stilling, Seth R. Bordenstein, Timothy G. Dinan, John F. Cryan

**Affiliations:** ^1^Alimentary Pharmabiotic Centre, University College CorkCork, Ireland; ^2^Department Anatomy and Neuroscience, University College CorkCork, Ireland; ^3^Departments of Biological Sciences and Pathology, Microbiology, and Immunology, Vanderbilt UniversityNashville, TN, USA; ^4^Department of Psychiatry, University College CorkCork, Ireland

**Keywords:** microbiota, sociality, neurodevelopment, gene-environment interactions, non-coding RNA, epigenetics, evo-devo, transgenerational

## Abstract

The tight association of the human body with trillions of colonizing microbes that we observe today is the result of a long evolutionary history. Only very recently have we started to understand how this symbiosis also affects brain function and behavior. In this hypothesis and theory article, we propose how host-microbe associations potentially influenced mammalian brain evolution and development. In particular, we explore the integration of human brain development with evolution, symbiosis, and RNA biology, which together represent a “social triangle” that drives human social behavior and cognition. We argue that, in order to understand how inter-kingdom communication can affect brain adaptation and plasticity, it is inevitable to consider epigenetic mechanisms as important mediators of genome-microbiome interactions on an individual as well as a transgenerational time scale. Finally, we unite these interpretations with the hologenome theory of evolution. Taken together, we propose a tighter integration of neuroscience fields with host-associated microbiology by taking an evolutionary perspective.

## Introduction

“NOTHING IN BIOLOGY MAKES SENSE EXCEPT IN THE LIGHT OF EVOLUTION”

(Dobzhansky, 1973)

It is now over 40 years since Dobzhansky published his famous essay aimed at defending evolutionary thinking against the increasing influence of creationist belief (Dobzhansky, 1973). Today the foundation of the neo-Darwinian synthesis that Dobzhansky helped engineer has grown into one of the central pillars of modern biology and underlines the essentiality of considering biological processes from an evolutionary viewpoint.

On inspection of the evolutionary and genomic trajectories of macroscopic species, biologists increasingly find that they are not only determined by changes in gene frequencies in the nuclear and cytoplasmic genomes, but also strongly by genetic variation in the single-celled symbionts that can be understood as part of the total genetics of the macroscopic organism. In this light, intergenomic interactions between the nucleus and microbiome are similar to the intergenomic interactions between the nucleus and the mitochondria, or even between chromosomes of the nucleus. Indeed since their origins, eukaryotes and their microbial symbionts have been and are being united in diverse associations, ranging from obligate intracellular to extracellular microbes that forge mutualistic, commensal and parasitic interactions (Dale and Moran, 2006; Dethlefsen et al., 2007; McFall-Ngai et al., 2013; Douglas, 2014). These diverse associations serve as raw genomic variation for natural selection to operate on. Just as a gene-gene interaction (e.g., epistasis) emerges from mutational events in the genome and can be selected upon, so too are symbiont-host associations formed and forged over time.

With the advent of multicellularity and mobile animals, energy demands rose and opened the door for microbial symbioses to intensify their role in host nutrition and metabolism. Still today, microbial influences continue to shape eukaryotic and animal evolution. The microbiota affects nearly every aspect of animal fitness as they colonize animal organs including the mouth, skin, reproductive organs or specialized organs, such as female reproductive tissues (Funkhouser and Bordenstein, 2013), the light organ of the Hawaiian bobtail squid *Euprymna scolopes*, and other surface organs exposed to the environment, with the gastrointestinal tract reaching the highest densities of bacterial cells in mammals (Turnbaugh et al., 2007; Dave et al., 2012; Schloissnig et al., 2013). Taken together, it is important to note that the universality of symbiosis in eukaryotic evolution does not obviate canonical mechanisms of evolutionary biology such as natural selection or even other levels of selection such as selfish genes. Rather, natural selection at multiple levels and symbiosis are operating together in underappreciated ways that are borne out as the microbiome sciences mature.

The host-associated microbiota is not only comprised of bacteria, but also archaea and eukaryotes such as protozoa, fungi and nematodes. Furthermore, viruses of all three cellular domains, collectively termed the virome, can be found in the microbiota (Virgin, 2014). Large-scale microbial sequencing projects like the Human Microbiome Project (HMP) (Turnbaugh et al., 2007; Human Microbiome Project Consortium, 2012), the European MetaHIT (Qin et al., 2010), and the Eldermet project (defining the microbial composition associated with aging, Claesson et al., 2012) have contributed to identify the human-associated microbiota, consisting of at least 40,000 bacterial strains in 1800 genera (Luckey, 1972; Frank and Pace, 2008; Forsythe and Kunze, 2013), which collectively harbor at least 9.9 million non-human genes (Li et al., 2014). Carrying approximately 500 times the human protein-coding genes currently annotated (http://www.ensembl.org), the ~100 trillion non-human associated cells make up 1–2 kg in an adult body (Forsythe and Kunze, 2013), which is comparable to the weight of the adult human brain (ca. 1.5 kg, Parent and Carpenter, 1996).

The above comparison ending in the analogy to the weight of the brain is not an arbitrary exercise in numbers. It is a window into the connections between neuroscience and microbiology. During human evolution, the primate brain underwent structural reconstructions of fast and dramatic increases in relative volume, leading to the brain as the most energy-demanding organ in the body (Khatri and Man, 2013). Interestingly, it has been observed that at the same time, the gastrointestinal tract shrunk accordingly, which led to the “expensive-tissue hypothesis” (Aiello and Wheeler, 1995), proposing compensation of growth of one metabolically expensive organ by reduction of another. While this latter hypothesis has been challenged (Navarrete et al., 2011; Warren and Iglesias, 2012), recent evidence suggests that the presence and types of microorganisms in a given host individual not only has multiple, critical consequences for host physiological processes such as postnatal development and immunomodulation, but also surprisingly affects neurodevelopment, host behavior and cognition (Cryan and Dinan, 2012).

In light of this new evidence and data from cognitive neurogastroenterology studies (e.g., Cryan and O'Mahony, 2011), we will explore the possibility that host-microbe associations critically affect mammalian brain evolution and development. We will further argue that to understand transgenerational inter-kingdom communication and their effects on brain adaptation and plasticity, it is inevitable to consider epigenetic mechanisms as important mediators of these host-microbe interactions.

## Genome-microbiome interactions and animal evolution

“THERE IS A FUNDAMENTAL ERROR IN SEPARATING THE PARTS FROM THE WHOLE, THE MISTAKE OF ATOMIZING WHAT SHOULD NOT BE ATOMIZED. UNITY AND COMPLEMENTARITY CONSTITUTE REALITY”

(Werner Heisenberg, 1930)

According to long-standing dogma in biology, a mammal's first contact with bacteria occurs during delivery through the birth canal. However, there is an increasing body of evidence that demonstrates maternal transmission of certain microbes occuring *in utero*, and thus the sterile-womb paradigm is out-dated (Funkhouser and Bordenstein, 2013). Moreover, the mother's gut microbiota changes dramatically during pregnancy (Koren et al., 2012). After delivery through the birth canal, the microbiota becomes more complex and abundant, and these community-level changes continue via breast-feeding and uptake of new microbes from the environment (Koenig et al., 2011). It is therefore not surprising that the microbiota critically influences pre-, peri- and postnatal development, and changes during early life stages will result in phenotypic alterations in adulthood (Borre et al., 2014). Moreover, microbial successions during animal development are well-established (Koenig et al., 2011; Brucker and Bordenstein, 2012b; Pantoja-Feliciano et al., 2013) and can be influenced by various environmental factors such as diet, lifestyle or habitat (Marques et al., 2010).

Microbiota colonization can depend on the genetics of the host, and there is an intensifying interest today in resolving the relative contributions of the environment and host genes on the assembly of host-associated microbial communities. In particular, the host genome may filter environmental microbes into host tissues as a form of symbiont domestication each generation, and reciprocally, environmental microbes may prefer to occupy specific lineages of hosts (Brucker and Bordenstein, 2012a). Several studies have explored genome-microbiome associations (Dethlefsen et al., 2007; Arumugam et al., 2011; Moeller et al., 2012), and laboratory studies in model systems are beginning to be utilized to control the influences of the macro- and microenvironment on gut microbiota assembly, thereby leading to an excavation of the intrinsic host-genetic influence on the assembly of the microbiota across species. Differential microbial compositions occur between closely related species when maintained on the same diet and under identical rearing conditions (Brucker and Bordenstein, 2012b; Franzenburg et al., 2013), and the community relationships of each species' microbiome parallels the phylogenetic relationships of the host genome, a pattern termed “phylosymbiosis” in the insect model *Nasonia* (Brucker and Bordenstein, 2013b). Moreover, in the early branching metazoan *Hydra* (a cnidarian that reproduces asexually), specialized anti-microbial peptides partly regulate phylosymbiosis across related species (Fraune and Bosch, 2007; Franzenburg et al., 2013). It has also been demonstrated that interspecific host-microbe specificity is required for proper immune system development in mice (Chung et al., 2012).

Within species, there are differences in the microbiome that can be attributed to single-nucleotide polymorphisms (SNPs) or copy-number variations (CNVs) (Rausch et al., 2011; Tong et al., 2014; Wacklin et al., 2014). As such, genetic variation between mouse strains is responsible for variations in gut microbiota (Benson et al., 2010; Kovacs et al., 2011). In humans, there is evidence that microbiota composition is more similar in closely related individuals such as monozygotic twins (Zoetendal et al., 2001) and correlates with ethnic affiliation (Ravel et al., 2011; Mason et al., 2013). Understanding the interplay between colonization dynamics of microbes, human genetics, and complex diseases, including neurodevelopmental and psychiatric diseases (i.e., autism spectrum disorders (ASDs), schizophrenia and depression), is an important endeavor to ultimatley define genetic risk factors for a potentially fatal microbial composition (Spor et al., 2011). In general, genotype-enterotype interactions may be a key determinant of microbial variation between individuals. With the progressing trend toward fecal transplantation treatments in mind (Borody and Khoruts, 2012), which is now even feasible from frozen fecal suspensions that can be deposited in specific fecal bio-banks (Youngster et al., 2014), incompatible or otherwise detrimental host genome-microbiome combinations should also be considered and donor-recipient pairs screened accordingly prior to fecal transplantation.

In conclusion, host selection of the microbiota exhibits features of an extended phenotype encoded by the core genome. The “extended phenotype” concept was introduced by Richard Dawkins to include modulatory effects on the environment as part of a gene's phenotype that extends beyond modulation of the cells in which it is expressed (Dawkins, 1983). As the phenotypes that we are discussing have genomes themselves, the analogy is more appropriately extended to an extended genome that encodes the essential features missing in the host genome. According to this perspective, symbiosis in general and microbial endosymbiosis in particular can be viewed as the essential complement of the missing activity of an organism's core genome, a view that is compatible with the hologenome concept, first introduced by Rosenberg et al. (2007). This concept considers the host's genome and it's associated microbiome as an organism's total genome, in which the summed genetics of all members can affect fitness and is thus a newly appreciated unit of selection that affects adaptation and speciation (Rosenberg et al., 2007; Brucker and Bordenstein, 2013a). Therefore, the hologenome concept embraces the contemporary gene-centric view of life, but upgrades it to include the microbiome as a central facet of an organism's genetics. By all accounts, this viewpoint blurs the differences between the genome and environment. It embraces a vibrant and more satisfying view of the nature of biology, namely that the microbiome is as essential as the genome in defining what an animal or plant is and is not.

## The social network: microbiota, RNA and the evolution of the social brain

“LIFE DID NOT TAKE OVER THE GLOBE BY COMBAT, BUT BY NETWORKING”

(Margulis and Sagan, 1986)

### Minds that think alike: advantages of social brains

Many mammalian species have evolved to give up solitary life and form social groups of cooperative individuals. Group living offers a variety of advantages ranging from mutual protection to cooperative foraging for food and finding a mating partner. Also support during rearing of offspring is likely to exhibit an evolutionary advantage in family-like structured groups as predicted by kin selection theory, a mathematical framework providing an explanation for the apparently paradox altruistic investment of resources to help nurturing offspring of close relatives (Hamilton, 1964). However, group living also poses certain challenges on the physiology and behavior of individuals within a social group. As such, group members need to recognize each other, which demands interaction of visual and memory systems in the brain. In order to plan and organize cooperative undertakings such as hunting, as observed in group-living mammals like wolfs, lions and some primates, also involves an acknowledgement of what other individuals know, see or feel. This form of empathy or “Theory of Mind” is a feature that is highly developed in humans and commonly disturbed in neurodevelopmental disorders of the autistic spectrum (Baron-Cohen, 2009). Moreover, neurobiological mechanisms for behaviors such as those related to affection need to be implemented in the brains of social individuals in order to secure social bonding within a group.

Therefore, the brains of social species exhibit a set of common features that need to work together for group living to become advantageous. Brain areas such as the prefrontal cortex or the amygdala have undergone pronounced changes in the evolution of social mammals such as humans (Hrvoj-Mihic et al., 2013). Also neuroendocrine systems, such as vasopressin and oxytocin, the latter of which is sometimes referred to as the “social hormone,” is important for group living to support affection and empathy among group members (Insel, 2010; Meyer-Lindenberg and Tost, 2012; Lukas et al., 2013; Skuse et al., 2014). Furthermore, it is increasingly appreciated that the social environment is tightly associated with susceptibility to mental health problems, with city-living being more detrimental than rural close-knit communities (Lederbogen et al., 2011).

Relationships to other individuals of the same species as well as behavior toward other species are also a key environmental factor influencing the specific microbiota of an individual. Social isolation or maternal neglect can lead to severe stress-related disturbances of the gut microbiota, which potentially mediate further adverse physiological reactions associated with stressful situations (Bailey and Coe, 1999; O'Mahony et al., 2009; Bailey et al., 2011). In fact, also from the reverse perspective, microbiota and social life may be more intimately connected than generally appreciated.

### Expanding the gene pool: the role of microbes in the evolution of the social group living

**Figure 1:**
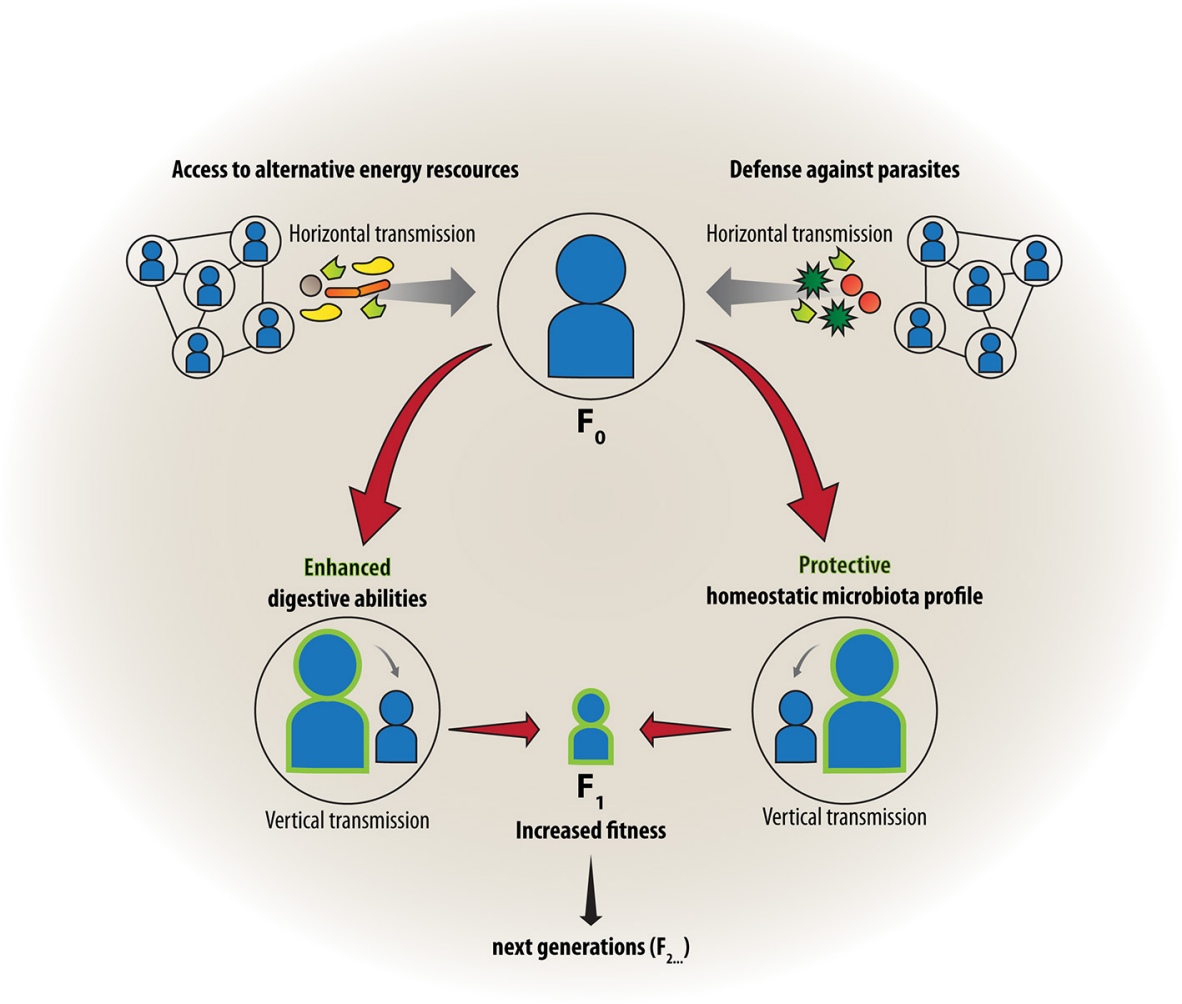
**Friends with benefits: Social group living and transmission of microbes**. Advantages of living in groups may occur through horizontal transfer of beneficial symbionts that increase abilities to digest a wider range of foods **(left)** or confer resistance against infectious agents **(right)**. Protection from parasites was suggested to be especially important for non-herbivores, where beneficial microbes create a healthy homeostatic microenvironment and train the immune system of the host. The acquired microbes can further be transmitted vertically to the next generation, strengthening the symbiotic association between host and microbe and conferring increased biological fitness.

Unlike our core genome, our microbiome is contagious. Microbial cells can actively or passively leave the body and spread to new habitats and hosts, and from an evolutionary perspective it can be assumed that natural selection has favored those microbes that increase their own transmission. On the other hand, exchange of microbes by intimate contact with conspecifics may offer benefits for the host as well. These features of the microbiome have motivated some authors to hypothesize that social behavior has, at least in part, evolved to enhance transmission of microbes (Troyer, 1984; Lombardo, 2008; Ezenwa et al., 2012; Montiel-Castro et al., 2013). As such, it can be an advantage to transfer beneficial symbionts, which are used by recipients either to increase resistance against infectious agents and toxins or increase abilities to digest a wider range of foods (Figure 1, left side). Lombardo has referred to this “access to mutualistic endosymbiotic microbes” as a driving force in the evolution of sociality in animals (Lombardo, 2008). While the microbe-dependent ability to process plant-based diets has been especially important for herbivorous species (Troyer, 1984), protection from parasites and pathogens was suggested to be driving social behavior in non-herbivores (Lombardo, 2008). The latter is likely achieved by exchange of microbes that contribute to the secondary metabolism by producing toxins or antibiotics that help to provide a defense against parasites or degrade toxic xenobiotics and create a healthy homeostatic microenvironment (Douglas, 2014) (Figure 1, right side). Furthermore, some microbial components such as bacterial cell wall material or other microbe-associated molecular patterns (MAMPs), (Ausubel, 2005) are recognized by the immune system and thereby prime it for encounters with pathogenic or unwanted microbes (Chung et al., 2012; Lee and Mazmanian, 2014). Montiel-Castro et al. further discussed the evolutionary role of specific social behaviors such as kissing, grooming and sexual intercourse for enhancing the transfer of microbes for selective colonization (Montiel-Castro et al., 2013) and just recently focused on the role of microbes in social and socioeconomic decision making (Montiel-Castro et al., 2014). Although the social-behavior-driven mechanisms of exchange of symbionts will also always be subject to hijacking by parasites, we must assume that the exchange of beneficial microbes prevails, considering the fact that there was never an option for evolution to select against all symbionts.

These notions are particularly intriguing in the light of British anthropologist Robin Dunbar's social brain hypothesis. This assumes a causal positive relationship between neocortex size and social behavior within primates as a factor critically contributing to evolution of human intelligence (Dunbar, 1998). It is therefore tempting to speculate that enhanced transmission of microbes through group living may have contributed to the gradual increase in cortical size and function. Studies investigating divergence of the microbiome along evolutionary speciation trajectories would need to show matching host and symbiont phylogenies to help inform such a hypothesis (Dale and Moran, 2006; Zaneveld et al., 2008; Fitzpatrick, 2014). A recent study comparing the chimpanzee and human microbiomes is a first step in this direction (Moeller et al., 2012) and makes it particularly intriguing for comparative biology to embrace microbiota differences that facilitate phylosymbiosis with regard to social complexity.

### Regulatory RNA networks

Tightly associated with the accelerated expansion of the neocortex during primate evolution, the human genome has seen accelerated evolution, especially in certain non-protein-coding regions. With the advent of whole-transcriptome sequencing technologies, we learned that a lot of these regions actually do contain valuable information. Interestingly, most of these regions are transcribed into RNA, albeit with often unknown or unassigned function. Yet, there is a steadily increasing amount of evidence that these non-coding RNAs (ncRNAs) have a potent regulatory impact on the cell's transcriptional landscape. They are grouped as small (<200 bp) and long-non-coding RNAs (>200 bp). Small RNAs have been established early in evolution (also prokaryotes are known to use them; Liu et al., 2012; Mika and Hengge, 2013) and are further divided into different subgroups, most importantly microRNAs (miRNAs), small-interfering RNAs (siRNAs) and piwi-interacting RNA (piRNAs), which together function in post-transcriptional regulation of gene expression by interfering with primary transcripts. Long non-coding RNAs (lncRNA), however, appear much later in evolution and are only found in plants and animals. In fact, about one third of the known lncRNAs seem to be primate-specific (Derrien et al., 2012; Barry, 2014). It is important to note that the brain is not only the main site of expression of lncRNAs but also of other RNA-based regulatory mechanisms, including alternative splicing, RNA editing and RNA methylation (Paul and Bass, 1998; Meyer et al., 2012; Li et al., 2013; Niu et al., 2013), most of which have greater prominence in the human genome, are particularly prevalent in the human brain, and are therefore promising candidates for a key role in the evolution of neurodevelopmental processes and complex human social behavior and cognition (Blow et al., 2004; Xing and Lee, 2006; Lin et al., 2010; Barry and Mattick, 2012; Qureshi and Mehler, 2012).

**Figure 2:**
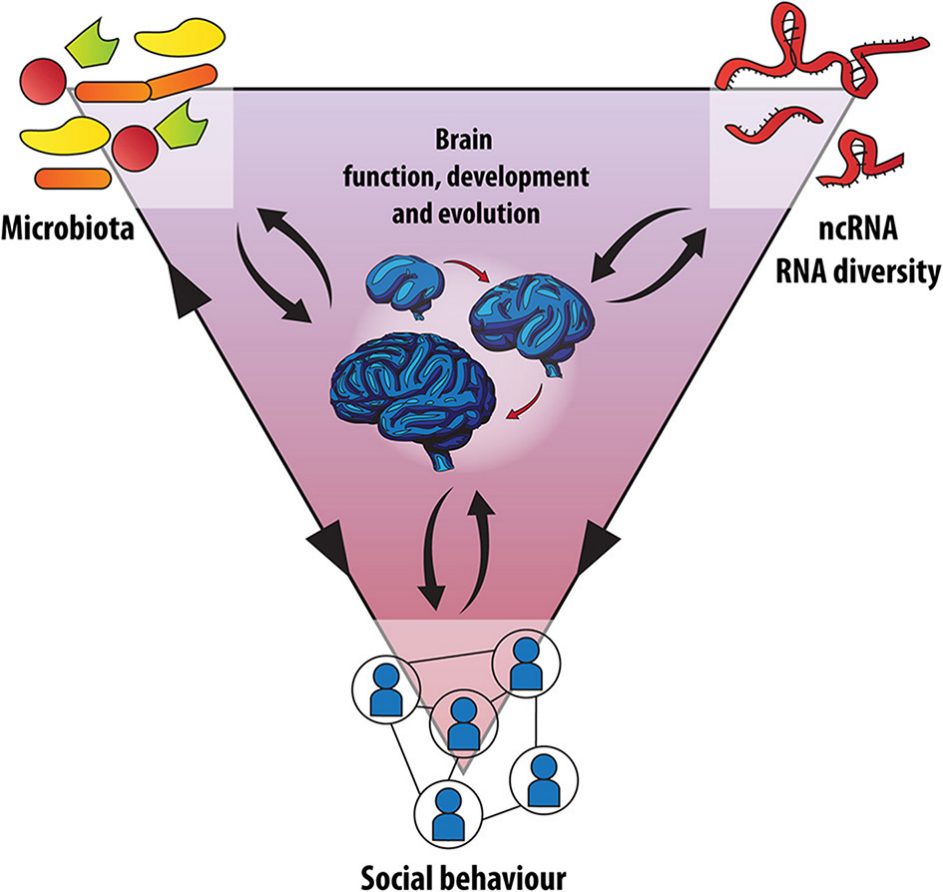
**Microbes, RNA networks and brain development: A social triangle?** An integrated model is proposed for the evolution of human social behavior. Recent human evolution was accompanied by accelerated extension of the neocortex along with an increase in the importance of ncRNAs and RNA diversity, processing and plasticity in the brain. At the same time host-microbe co-evolution contributed to enhance sociability by providing endosymbiotic developmental signals through the microbiota-gut-brain axis. In addition, social behavior affects the composition of the microbiota and *vice-versa* and differential expression of ncRNAs has been observed in cognitive disorders that are associated with altered social behavior (black arrow heads). Whether the microbiota and the brain's transcriptome interact, especially on the level of non-coding RNAs, is currently under investigation.

Together, both enhanced microbial transmission through group living and expansion of non-coding RNA regulation, likely have contributed to advanced social behavior in primates and ultimately human intelligence (Figure 2). However, it will be intriguing to understand whether these two systems developed in parallel and independently, or whether host-symbiont co-evolution had an impact on the development of more complex RNA regulation and *vice versa* (Figure 2). Based on this, we predict that dysbiosis during development or the complete absence of microbes during germ-free life leads, among other effects and through mechanisms that remain to be identified, to a deregulation of key RNA-based processes necessary for normal brain maturation and function.

### Microbiota, RNA networks and brain development: a social triangle?

In order to interrogate a potential interaction between RNA networks and the microbiota in neurodevelopment and evolution of social behavior, it will be helpful to look at two kinds of experimental contexts. Firstly, scenarios where disturbances of social capabilities of a host need to be investigated with respect to changes in the microbiota and RNA-based gene regulation. Secondly, effects of the absence of microbes or of a disturbed microbiota composition (dysbiosis) on RNA-based mechanisms *and* social behavior will be crucial in informing the genetic basis of this social triangle (Figure 2).

With respect to the former, among the most pronounced disturbances of social and emotional behavior are disorders within the autism spectrum. On the one hand, ASDs and other neurodevelopmental disorders have recently been associated with impaired function of ncRNAs (Mellios and Sur, 2012; Van De Vondervoort et al., 2013; Ziats and Rennert, 2013). Moreover, on the other hand, recent research suggests that neurodevelopmental disorders, such as ASDs, are tightly interleaved with the gut microbiota. Though it is unknown at the moment what is cause and what consequence, the most common symptom of ASDs—a lack of pro-social behavior such as sociability—is often accompanied by gastrointestinal (GI) symptoms and alterations in microbiota composition and function (Ming et al., 2012; Cao et al., 2013). Interestingly, both, CNS and GI symptoms are also commonly co-occurring in animal models for ASDs and seem to be dependent on host-microbe interactions (Hsiao et al., 2013; Desbonnet et al., 2014).

Some correlative evidence for an interaction of the microbiota and RNA editing comes from a study in genetically obese leptin-deficient (*ob/ob*) mice, which are known to harbor a different microbiota compared to lean mice (Turnbaugh et al., 2006), and show altered editing of the serotonin receptor *Htr2c* mRNA in the hippocampus and hypothalamus (Schellekens et al., 2012). Interestingly, these mRNA-editing sites are precisely conserved in humans and alterations in editing frequency has been shown to be associated with schizophrenia (Burns et al., 1997; Dracheva et al., 2003), although regulation in humans happens to be more complex due to alternative splicing of the editing cassette. Also RNA methylation might be connected to microbiota composition through metabolism, since one of two known RNA demethylases, the *Fat mass and obesity associated (Fto)* gene, was found to be strongly associated with body mass index in humans (Speakman, 2013; Zheng et al., 2013).

In order to take a closer look at the second experimental context, i.e., the effects of the absence of microbes on social capabilities, we have recently shown that germ-free mice largely lack pro-social behavior and social cognition (Desbonnet et al., 2014). Germ-free mice spent significantly less time with a conspecific and did not show the typical preference for a novel mouse when given a choice between a familiar and a novel interaction partner. While sociability could be rescued by introduction of a normal microbiota post-weaning, recognition memory was not amenable by microbiota replenishment, suggesting a critical developmental time window for microbiota-dependent cues to act upon the central nervous system and the establishment of social abilities (Desbonnet et al., 2014). Decreased sociability, albeit less pronounced compared to the mouse model, was also demonstrated in germ-free rats (Crumeyrolle-Arias et al., 2014). Future experiments should also focus on dynamic regulation of gene expression and especially the role of regulatory RNA in these animals, with a focus on brain regions involved in social behavior.

Taken together these concepts support a model in which the evolution of human sociability, which was accompanied by accelerated extension of the neocortex, is a key example of host-microbe co-evolution, and is dependent on endosymbiotic developmental signals through the microbiota-gut-brain axis (Figure 2). The development of the forebrain, esp. the neocortex, in social mammals and ultimately primates and humans depends on correct and timely signals from microbial symbionts—which is disturbed, when the microbiota is absent or disturbed, as seen in artificial germ-free models or in more natural settings such as cesarean section or pre-, peri- or early life stress (Gilbert et al., 2010; Borre et al., 2014).

But what are these environmental cues that the microbiota provide for host brain development? And how can they interfere with RNA-based mechanisms? In the following sections we will review evidence that, in addition to modulation of neurotransmitter systems, epigenetic mechanisms of gene regulation provide a suitable interface for host-microbe interactions. To do so, we will first explore how epigenetic mechanisms shape Darwinian evolution and then focus on the molecular epigenetic machinery that provides an interface for interaction with such mechanisms for the microbiota to affect evolution and development of the social brain.

## Epigenetic mechanisms in evolution: nature and nurture

“EPIGENETICS IS A USEFUL WORD IF YOU DON'T KNOW WHAT'S GOING ON – IF YOU DO, YOU USE SOMETHING ELSE.”

(Adrian Bird, 1995)

In addition to microbe-induced genetic variation such as lateral gene-transfer (LGT, see Box 1), symbiont-dependent *epi*genetic mechanisms can generate heritable variety within a few generations, which may reduce the amount of germline LGT needed to make symbiont-induced changes to host development on short time scales.

Box 1 **Lateral gene transfer (LGT) as a source of selectable variability.**

Interdependencies between species do not only occur between host and microbes but also among the individual microbes, namely through metabolic chains and genomic complementarity, in which metabolites and gene products are exchanged to fulfill those missing from the interacting partner(s). In addition to chemical or peptide exchanges, genetic material can also be swapped between microbe and host. From the perspective of the hologenome theory, host-associated microbes represent a continuum of symbiotic interactions that scale from bacteria-derived organelles to endosymbionts and extracellular microbes. Across the developmental trajectory of early eukaryotes, the bulk of chloroplast and mitochondrial genes have been relocated to the nuclear genome. While the interactions between eukaryotic nucleus and extracellular microbes are certainly less intimate, it has to be noted that interspecies lateral gene transfer (LGT, also known as horizontal gene transfer) is continuing to occur also between eukaryotic and viral as well as prokaryotic genomes (Salzberg et al., 2001; Robinson et al., 2013; Overballe-Petersen and Willerslev, 2014). It is clear that viral genes strongly contributed to animal genome evolution and fitness. Up to 8% of the human genome is estimated to be of viral origin (Belshaw et al., 2004), followed by another 37% sharing homology with bacterial genes (McFall-Ngai et al., 2013), though it is still unclear if the majority of these bacterial genes have been transferred to animal genomes by LGT or stem from early eukaryotic evolution (Domazet-Loso and Tautz, 2008).

Inter-microbial LGT is common and particularly (in)famous for the quick spread of antimicrobial resistance. Interestingly, it can also serve as a relatively quick response toward a changing gut environment, e.g., during gut inflammation (Stecher et al., 2012). Compared to bacteria-to-bacteria transfer, LGT between animal hosts and microbes is less common (Blaxter, 2007). This is mostly due to the fact that LGT has to occur, at least in sexually reproducing animals, in the germ cells to be transmitted to the next generation and be stabilized in the population (Robinson et al., 2013). However, recently these events are identified more frequently, especially in invertebrates (Boto, 2012), some of which are tightly associated with the germline-transmitted bacterial endosymbiont Wolbachia (Dunning Hotopp et al., 2007). By modifying an organisms DNA, LGT provides a source of selectable genetic variation over time in addition to base pair mutations, recombination, insertions, deletions, etc., and may therefore act as an effective driver of co-evolution, especially on longer evolutionary time scales.

Initially the term “epigenetics” was used to describe developmental programming (Waddington, 1953), and was only later defined to refer to heritable changes in gene expression that do not originate from mutations of the DNA sequence (Holliday, 1987). More recently, the definition of the term is discussed frequently and controversially (as outlined by the initial quote found in Ledford, 2008; Ptashne, 2013a,b) and now often used in a much broader sense, with specific connotations depending on the field of study. In contrast to the former strict definition, some researchers focus on the aspect of sequence-independent transgenerational germline inheritance of a phenotypic trait, especially neuroscientists or biological psychiatrists, who highlight the impact of early-life experiences on development and behavior during later life (e.g., Meaney and Szyf, 2005). Yet another branch of developmental biology interprets transgenerational epigenetic traits in the context of cellular differentiation during multicellular organismal growth (somatic mitotis; Müller and Leutz, 2001; Steffen and Ringrose, 2014). Most of these definitions have in common one or the other aspect of (transcriptional) memory in the sense that the effect of a stimulus is perpetuated even if the initial signal or event disappears (Ptashne, 2007). Inspired by an apparent overlap of this with memories in the brain, the term epigenetics in molecular neurobiology is often used rather loosely to point to a common set of molecular signaling cascades affecting dynamic regulation of gene expression due to neuronal activity, also referred to as neuroepigenetics or chromatin plasticity (Dulac, 2010; Sweatt, 2013; Fischer, 2014a).

Some recent studies have pointed to the intriguing possibility that life experience and other environmental insults acquired in the parental generation may result in altered brain function and behavioral changes in subsequent generations (Weaver et al., 2004; Arai et al., 2009; Franklin et al., 2010; Bohacek et al., 2013; Dias and Ressler, 2014; Gapp et al., 2014). While only few studies showed that supportive influences such as enhanced memory function through environmental enrichment (Arai et al., 2009) or avoidance of a potentially harmful odor (Dias and Ressler, 2014) can be passed on to the next generation, this epigenetic transgenerational inheritance is best documented for rather stressful events such as exposure to trauma. This bias might be explained by easier access to experimental models that induce robust and functional epigenetic changes that have negative outcomes such as increased depressive or anxiety-like behaviors than appetitive-based environmental situations. Additionally, epigenetic transgenerational inheritance might have evolved to provide offspring with protection against rapid, adverse changes in the environment of the parental generation and serve as means to quickly adapt to current environmental situations. They can thus be interpreted as mechanisms that offer evolutionary “short-cuts” to circumvent the long process of sustained natural selection, which is needed to represent environmental information in the DNA sequence of the genome, and be able to respond to unpredictable, rapid changes in the environment that occur within the life-time of a generation. Interestingly, in experimental models, the behavioral effects of these epigenetic adaptations quickly wear off if not reinforced, which underlines their plasticity and reversibility. This impermanence also highlights that there is little need for some epigenetic traits to be stabilized in a population and become part of hard-wired Mendelian mechanisms of inheritance, since the environmental factors leading to these traits are dynamic and unpredictable.

However, if the effects of traumatic or stressful experiences can be inherited to prepare and protect offspring from a harmful environment, it raises the question whether the observed behavioral phenotype in experimental models, including depressive- and anxiety-like behavior, are in fact protective. If these changes are not just pleiotropic by-products of the epigenetic machinery regulating gene expression in the parent, then there must be a beneficial effect of passing them on to the next generation and the behaviors expressed by the offspring may actually be protective. While we have little reservation in accepting that useful information about current conditions should be (epigenetically) transmitted to the next generation, there is some difficulty in understanding the benefit when adverse conditions trigger negative effects such as increased risk for psychiatric disorders in subsequent generations. However, there could be scenarios in which it would be beneficial to inherit information about environmental challenges that negatively affected the parental generation and will continue to affect offspring.

One such situation proposes that negative effects, such as the physiological and behavioral responses to stressors that are observed in experimental setups, are not *negative* to begin with. Consequently, increased stress-responsiveness, decreased resilience or depressive-like behavior may actually be protective traits under the conditions experienced by the parental generation. This reasoning is in agreement with the interpretation of the physiological stress response that Hans Selye advocated when he first published the stress theory (Selye, 1978; Dubrovsky, 2002) and that was later reused by Munck et al. (1984) to underline the protective function of elevated glucocorticoid levels in response to stress. Interestingly, this interpretation also offers an explanation of the apparent “corticosterone-paradox” observed in germ-free mice: While corticosterone levels are generally higher in germ-free mice at baseline and even more exaggerated under stress conditions, they actually show decreased anxiety-like behavior. Accordingly, evolutionary psychiatric researchers such as Nesse (2000) or Stevens and Price (2000) argued for the hypothesis that depression and other psychiatric conditions may actually be evolutionary adaptations with a net increase in human fitness. However, this latter hypothesis has been heavily criticized with respect to the interpretation of evolutionary mechanisms (Dubrovsky, 2002; McLoughlin, 2002) and their relevance to clinical practice.

Alternatively, epigenetic inheritance of stressful events could be pleiotropic, i.e., they are negative side effects of otherwise beneficial mechanisms that provide flexibility and versatility in response to environmental challenges and usually confer an overall net advantage. Similarly, researchers such as Crow and Baron-Cohen advocate a role for pleiotropic effects in mental disorders with Crow suggesting that schizophrenia and psychosis may be atypical patterns of brain hemisphere lateralization and therefore a side-effect of genetic variation that was necessary for the evolution of human language (Crow, 1995, 1997) and Baron-Cohen proposing a theory of autism that explains ASDs as adverse effects of the development of higher cognitive function (“systemizing”) on the cost of empathy-supporting functions (“empathizing”) (Baron-Cohen, 2009). It is tempting to speculate whether also negative side effects of epigenetic inheritance are involved in these conditions.

While it is difficult to empirically test whether either or both of these scenarios are actually at the root of increasing risk for such disorders, these events likely reflect the costs of maintaining a beneficial epigenetic inheritance system that can sometimes go awry.

## The molecular epigenetic machinery: an interface for microbe-brain interactions in evolution and development

“THESE IDEAS ARE LIKELY TO HAVE PROFOUND CONSEQUENCES WHEN YOU START TO TALK ABOUT HOW THE STRUCTURE OF SOCIETY INFLUENCES COGNITIVE DEVELOPMENT. WE'RE BEGINNING TO DRAW CAUSE-AND-EFFECT ARROWS BETWEEN SOCIAL AND ECONOMIC MACROVARIABLES DOWN TO THE LEVEL OF THE CHILD'S BRAIN”

(Michael Meaney, 2006)

### Chromatin plasticity and non-coding RNAs in neuronal function and development

The gut microbiota produces many neuroactive compounds, which can directly affect how neurons communicate with each other. Among these are amino acids, (e.g., GABA and tryptophan), as well as monoamines, such as serotonin, histamine and dopamine, used as neurotransmitters in the brain or precursors of such (Lyte and Freestone, 2010; Lyte, 2011; Thomas et al., 2012a; Wall et al., 2014). In germ-free mice, dopamine and glutamate receptor expression (Sudo et al., 2004; Heijtz et al., 2011; Neufeld et al., 2011) as well as tryptophan metabolism and serotonin levels are significantly altered in the circulation but also in the brain during development (Clarke et al., 2013), suggesting that neurotransmitters and their precursors may be some of the cues that are provided by the microbiota to establish the gut-brain axis as an important regulator of neurodevelopment. However, there is accumulating evidence that also molecular epigenetic mechanisms are involved in shaping brain formation and functioning and that can be influenced by microbial symbionts.

**Figure 3:**
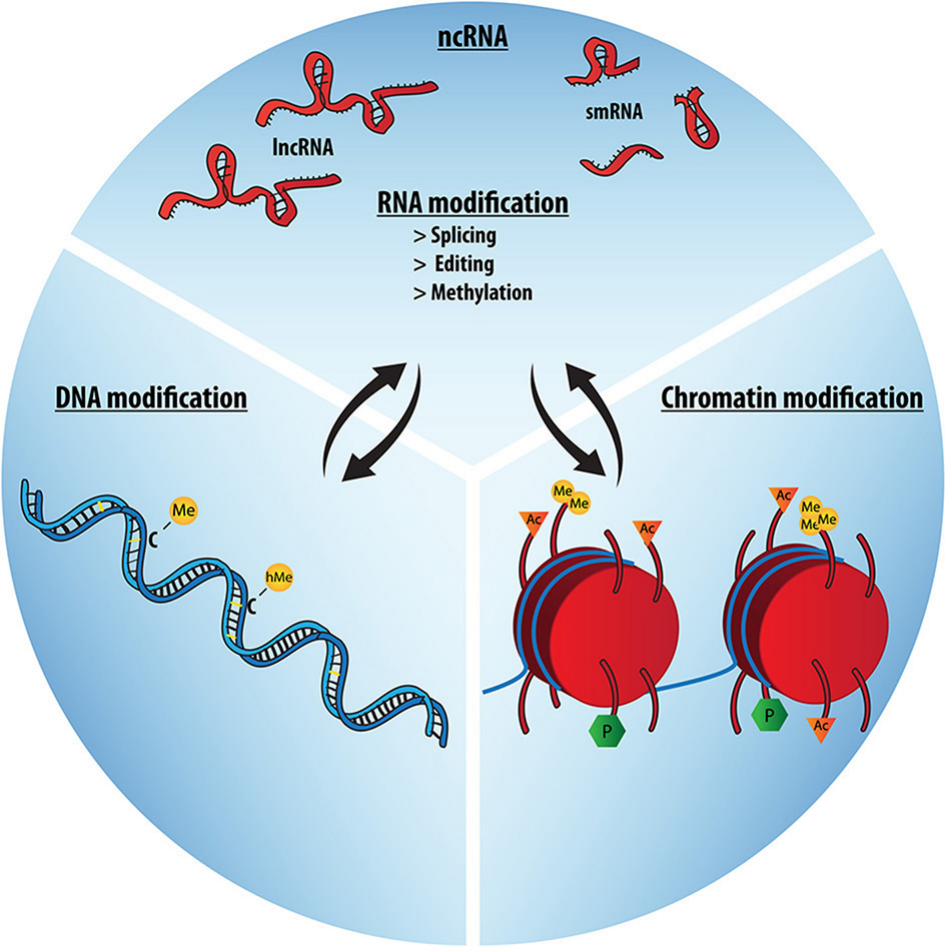
**The epigenetic trio: RNA, DNA and Chromatin modifications**. The molecular epigenetic machinery is comprised of DNA, chromatin modifications and several RNA-based mechanisms, most importantly various species of non-coding RNA. Together these mechanisms orchestrate developmental and gene expression patterns. They form a regulatory network, with significant interaction and mutual influences between the different domains (black arrows).

The question of which molecules and molecular mechanisms make up the epigenetic machinery follows the debate about its definition. Even if final proof about cause or consequence with respect to gene regulation is scarce, the majority of the literature now includes mainly three molecular mechanisms to constitute the molecular epigenetic machinery, mediating plastic changes in regulation of nuclear architecture, chromatin structure, and gene expression. Namely, these are histone modifications, such as acetylation or methylation, DNA modifications, such as CpG-methylation or –hydroxymethylation, and regulatory RNAs (Figure 3). These key regulators of gene expression integrate environmental signals and other stimuli at the transcriptional or translational level and can thereby lead to switching the amount of expression of a gene, which is an important factor exposing the effect of polymorphisms to the current environment. This makes epigenetic mechanisms important mediators of gene-environment and genome-microbiome interactions.

Importantly, all of these processes have also been shown to play an important role in cognitive function during health and disease (for recent reviews see Day and Sweatt, 2011; Kosik et al., 2012; Fischer, 2014a; Woldemichael et al., 2014). As such, long-term memory consolidation and synaptic plasticity greatly depend on dynamic regulation of gene expression in the hippocampus (Igaz et al., 2002, 2004; Da Silva et al., 2008) and roles and interactions of all three mechanisms of the epigenetic machinery have been identified. Histone acetylation is best known for its adjuvant role in learning-induced gene regulation. The catalyzing enzymes (histone acetyltransferases, HATs, and histone deacetylases, HDACs) are well-studied and can be targeted pharmacologically, which makes them promising targets for the treatment of neurodegenerative diseases and cognitive decline (Stilling and Fischer, 2011; Gräff and Tsai, 2013; Fischer, 2014b). The mechanism of action for these treatments supposedly involves the enhancement of the neuron's inherent response toward activation or facilitation of the normal regulatory program where it got out of balance (Peleg et al., 2010; McQuown and Wood, 2011; Stilling and Fischer, 2011).

More recently, also RNA-based plasticity is beginning to emerge as a crucial regulator of neuronal function, brain development, cognition and psychiatric disease (Barry and Mattick, 2012; Qureshi and Mehler, 2012). These include small RNAs such as miRNAs (O'Connor et al., 2012; Saab and Mansuy, 2014) and piRNAs (Landry et al., 2013) but also lncRNAs (Ng et al., 2013; Schaukowitch and Kim, 2014) as well as RNA editing, which primarily affects mRNAs expressed in the brain and has dramatically increased in humans compared to other species, and stimulus-dependent alternative splicing (Schor et al., 2009, 2013). Also mRNA methylation was recently reported to be enriched in brain tissue and considerably increased in the adult brain (Meyer et al., 2012). Importantly, many of these RNA-based processes are not only interacting with each other (Barry and Mattick, 2012) but are also intimately linked to other partners of the epigenetic machinery (Figure 3). For example, lncRNAs can act as sequence-specific guide molecules for histone-modifying enzymes, such as histone methyltransferases (Sanchez-Elsner et al., 2006; Rinn et al., 2007), and chromatin modifications help to determine splicing site selection (Luco et al., 2011; Kornblihtt et al., 2013), to name just a few.

### How can the microbiota act on development and behavior through epigenetic mechanisms?

The idea that epigenetic mechanisms could be key mediators of interactions between hosts and pathogenic or parasitic microorganisms was previously put forward (Minárovits, 2009; Paschos and Allday, 2010; Al Akeel, 2013; Silmon de Monerri and Kim, 2014; Stilling et al., 2014). However, it is clear that similar constructs apply to the interaction between host and non-pathogenic microbiota (Shenderov, 2012; Shenderov and Midtvedt, 2014) and that this may have strong implications for the regulation of brain evolution. In the first instance, it is important to detail some of the potential players in this context.

#### Short-chain fatty acids

In addition to synthesizing neurotransmitters or precursors, the gut microbiota produces other chemicals with neuro-modulatory potential. As such, fermentation of fiber by gut bacteria is the prime source for short chain fatty acids (SCFAs) such as butyric acid, propionic acid and acetic acid. SCFAs are not neuroactive substances *per se* but may act on neuronal function more subtly. For example, butyrate is best known for its potent inhibition of HDACs (Candido et al., 1978; Davie, 2003). While we recently reviewed details of the interactions of SCFAs with the epigenetic machinery elsewhere (Stilling et al., 2014), there is now solid new evidence that microbes do have a significant impact on epigenetic regulation in the host's gut epithelium and immune system. The effects were reported to be largely mediated by butyrate and related to altered HDAC activity (Alenghat et al., 2013; Arpaia et al., 2013; Furusawa et al., 2013; Smith et al., 2013; Chang et al., 2014).

In addition, acetic acid will affect the availability of HAT substrate (Acetyl-Coenzyme A) and thereby lead to higher levels of dynamic histone acetylation in neurons, which may be impaired when the normal microbiota is disturbed or completely absent. Together, both mechanisms will lead to increased histone acetylation, which facilitates memory consolidation, neurogenesis and neuroprotection (Fischer et al., 2007, 2010; Kilgore et al., 2010; Peleg et al., 2010; Govindarajan et al., 2011). Notably, intracerebroventricular infusions of propionic acid was shown to induce autistic-like behaviors in rats (MacFabe et al., 2011; Thomas et al., 2012b), suggesting SCFAs are also implicated in modulating social behaviors. Though the effects of SCFAs that cross the blood-brain barrier under normal conditions may be marginal, persistent secretion of SCFAs by the gut microbiota may result in cumulative, long-lasting effects on gene expression patterns that are necessary for appropriate neuronal development and function.

In conclusion, microbial SCFAs are important contributors to host metabolism and thus form a key part of the holometabolome acting as an energy source or through balancing host gene expression throughout brain development and, more dynamically, in adulthood (Selkrig et al., 2014).

#### Microbial mimicry of the host epigenetic machinery

Today, it is well-documented that microbes can directly target the host's transcriptional regulatory machinery. Especially viruses are known to harness infected cells for their own benefit, which comes to no surprise since they critically depend on their host's molecular machinery for replication and propagation. As such, certain influenza viruses use the host's epigenetic machinery to stimulate their own replication or hide within silenced regions of the hosts genome (Minárovits, 2009). Another mechanism to evade the host cell's antiviral response utilizes the virus-encoded histone-mimicking protein NS1 that mediates transcriptional repression (Marazzi et al., 2012). But also several bacteria can secrete proteins that mimic components of the host epigenetic machinery (Bhavsar et al., 2007; Murata et al., 2007; Hamon and Cossart, 2008; Pennini et al., 2010; Bierne and Cossart, 2012; Bierne et al., 2012; Rennoll-Bankert and Dumler, 2012; Bierne, 2013; Eskandarian et al., 2013; Rolando et al., 2013). Up to now, however, such effectors have been found exclusively in intracellular parasites like *Legionella pneumophilia*, which have direct contact to the intracellular environment to modulate host-cell transcription. In addition, RNA-based mechanisms targeting host transcriptional regulation have been added to the growing list of microbial effects on host transcription (Liu et al., 2012). This study found that *E. coli* is capable of producing short non-coding RNAs that act similarly to short interfering RNAs (siRNAs) on some host mRNAs. This interaction does not rely on intracellular localization of the symbiont, yet it is still unclear how the *E. coli* non-coding RNAs bridge the species barrier to interfere with *C. elegans* mRNA.

Most research in this regard has focused on parasites, i.e., viruses and bacterial pathogens (Minárovits, 2009; Paschos and Allday, 2010; Al Akeel, 2013; Silmon de Monerri and Kim, 2014), and it remains and open but intriguing question whether pathogens in the brain have means to alter transcription in neurons, which in turn could have an effect on host behavior. Most recently this prediction seems to have realized in the finding that *Toxoplasma gondii* infection results in DNA-hypomethylation at the promoter of the *arginine vasopressin (Avp)* gene and therefore increased expression of this gene in the amygdala of infected rats, which was sufficient for inducing the behavior-manipulation phenotype (Hari Dass and Vyas, 2014). Furthermore, it is unclear if similar mechanisms exist in any of the gut microbes. However, these pioneering studies demonstrate the various ways microbes use to interact with the host's epigenome.

In conclusion, there are several potential routes for microbes to interact with host cellular function and even behavior and some of these may be mediated by alterations of the epigenetic gene regulation in the brain.

## Confusing interactions – who is the puppet, who the puppeteer?

“MOST UNFORTUNATELY, IN THE LIVES OF PUPPETS THERE IS ALWAYS A *BUT* THAT SPOILS EVERYTHING.”

(Carlo Collodi, 1883, *The adventures of Pinocchio*)

Out of the 5 canonical ways different species can interact with each other (mutualism, commensalism, rivalry, predation and parasitism), parasitism long seemed to have the greatest impact on accelerating evolution. This has been acknowledged by the so-called “Red Queen Hypothesis,” a concept used to describe rapid host-parasite co-evolution by paraphrasing a character of Lewis Carroll's novel *Alice's Adventures in Wonderland*. Other forms of symbiosis, especially mutualism, have been neglected in this regard, although they now emerge to be just as important (Ezenwa et al., 2012).

However, in many cases it turns out to be challenging to determine where a given microorganism is located on what appears to be a mutualism-parasitism spectrum, unless the co-evolutionary trajectory is fully known. In addition, changes in the environment may revise the nature of the association. For example, it has been hypothesized that *Mycobacterium tuberculosis*, a well-known pathogen today, may have contributed to human evolution as a beneficial symbiont by providing a source of essential nicotinaminde during meat shortages (Williams and Dunbar, 2014). Further evidence for a strong dependency on environmental conditions determining how a particular microbe is associated with the host comes from a recent study on *Helicobacter pylori*, that appears to exhibit significantly differing virulence in two independent human populations (Kodaman et al., 2014). Thus, a causal timeline of symbiosis is hard to reconstruct in retrospective and even what appears as obligate mutualism today may not have started as mutually beneficial.

Classification of host-microbe interactions is further complicated by the need to define benefits and disadvantages for either side. As such, it is highly debatable whether strict commensalism actually exists. Also in mutualistic relationships, many cases may not be clear-cut. In such a relationship, both species mutually benefit from one another. For example, while the gut microbiota benefits from a constant supply of nutrients and a relatively high ambient temperature, allowing fast metabolic turnover, the host benefits from increased nutrient availability through enzymatic activity that is not coded in the host genome (e.g., digestion of fiber). But, since these advantages of the relationship may have compensated possible disadvantages during evolution, negative side effects of the association may be ignored or not recognized as detrimental in retrospect today.

In this respect, an exciting question regarding the evolution of social behavior is: Who is the puppet and who the puppeteer in social transmission of microbes? On the one hand, microbes that develop means of faster and more frequent transmission will be positively selected for. This may include co-evolutionary mechanisms and developmental cues, such as described in this article, which lead to increased sociability of a population or species or even direct behavioral manipulation of the individual host. This further relates to the question: What's in it for the microbiota? Why do they provide cues for development, why do they influence behavior? A recent attempt to answer some of these questions has been put forward, arguing that feeding behavior of the host might be manipulated by the special interests of particular microbes residing in the gut (Alcock et al., 2014). Another, simple but provocative explanation, derived from data discussed in this article, is that the bacteria promote social behavior in mammals and group living to more easily spread to new hosts and thereby reproduce more efficiently.

Indeed, fascinating examples of host-microbe interaction are found in parasites that are known to manipulate host behavior in order to reproduce and spread. Researchers find a growing variety of parasites in all domains of life that depend on altering host behavior for completion of their complex life cycles. These include members from a range of phyla such as the small liver fluke *Dicrocoelium dendriticum*, the zombie-ant fungus *Ophiocordyceps unilateralis*, or the Gordian worm *Paragordius tricuspidatus*, but also more common ones such as the rabies virus, which induces aggression as well as water avoidance behavior in mammals, or the ubiquitous protozoan *Toxoplasma gondii*, best known for inducing attraction of rodents toward cat urine scent, but also implicated in schizophrenia (Berdoy et al., 2000; Webster, 2001; Vyas et al., 2007; Libersat et al., 2009; Cézilly et al., 2010; Thomas et al., 2010; Flegr, 2013; Webster et al., 2013; for further reading see the special issue on neural parasitology in the January 2013 issue of the Journal of Experimental Biology). Interestingly, the underlying neurological mechanisms for these cases have yet to be revealed in detail. Given the very intimate contact with intracellular neuronal processes, it is intriguing to speculate that host-behavior alteration may be achieved by manipulating the transcriptional machinery of host neurons.

However, the answer to why a normal, healthy, and rather non-parasitic microbiota may provide developmental cues that facilitate social behavior may be a lot less spectacular than this. The problem is that we tend to think about this matter within a teleological framework and impose intentions on the bacteria. Furthermore, we tend to forget all eukaryotic evolution has always occurred in the presence of microbiota: Animals have never lived, and could never live, germ-free outside laboratory isolators. From this perspective the question is just the wrong question to ask and there is probably no satisfactory answer to it, because the microbiome just became part of multicellular bodies while these formed and now share a long history of co-evolution together. Given this tight association, it seems that we cannot simply impose opposing intentions for the microbiota and the host, since we also would not do this for genuine host-tissue cells.

## Conclusions & future perspectives

### The microbiota as “episymbionts” within a holobiont

“ALL EVOLUTION IS CO-EVOLUTION”

(Stuart Kauffman, 1995)

In this article, we present potential mechanisms for host-microbe interactions through molecular epigenetic processes and offer arguments to suggest that alterations of the microbiome and epigenetic modifications as well as RNA-based regulation of gene-expression are linked in shaping brain evolution and neurodevelopment. Apart from presenting evidence for rather direct influence on neuroendocrine and neurotransmitter systems, we further suggest that some microbial products can modulate the epigenetic landscape of the host brain. These possibilities include regulators of the activity of histone-modifying enzymes either through metabolic alterations or direct interactions between bacteria-secreted molecules (such as SCFAs) and host signaling pathways. Together these mechanisms provide important and time-critical cues for host neurodevelopment and thereby continue to influence social behavior in a co-evolutionary and co-developmental manner. At the moment, it is unclear if any of the behavioral phenotypes that are associated with altered microbial colonization, including models for ASDs, are due to decreased availability of SCFAs and/or other metabolites with epigenetic modifying function. Evidently, some parasites are able to highjack the host-cell's epigenetic machinery and it may prove worthy to look for similar effectors in symbiotic or commensal bacteria to assess translatable, therapeutic potential of such mechanisms.

Additionally, we argue that the microbiota is a key interface for gene-environment interactions. These interactions may lie at the heart of incompatible genotype-enterotype combinations and supposedly have critical consequences for the study of disease-associated genetic risk factors with yet unrevealed function. Such incompatibilities will also have implications for donor screening in fecal transplantation therapies.

With these functions in mind, we notice that the microbiota also shares some important characteristics in its interaction with the host that compare to classical epigenetic mechanisms such as histone modifications, DNA methylation and ncRNA-mediated regulation. These characteristics include (1) vertical transmission (transgenerational inheritance) of (acquired) microbes, (2) response to environmental stimuli and facilitation of gene-environment interactions, (3) determination of gene expression programs and developmental regulation, and (4) reversibility.

Hence, it is intriguing to look at the canonical epigenetic mechanisms as mediators of developmental signals sent by the microbiota as well as to also consider the symbionts themselves as epigenetic entities, as we and others have previously argued (Gilbert et al., 2010; Fitzpatrick, 2014; Stazi and Toccaceli, 2014; Stilling et al., 2014). However, the microbiome is clearly more complex and dynamic than an epigenetic signal since symbionts have their own genomes that can respond to adaptation where a molecular epigenetic modification itself is not subject to natural selection. Yet, while the analogy may not be perfect in details, this perspective is useful to understand how the microbiome represents a further interface for environmental influence and a dynamic source for transgenerational developmental regulation. It furthermore places the microbiota in line with other mechanisms that accelerate short-term environmental adaptation and may be especially helpful in unifying different theories of host-microbe co-evolution and the evolution of the “social brain.” In fact, this viewpoint may hold some answers to fundamental questions in the fields of epigenetics and neuroepigenetics (Bohacek et al., 2013; Sweatt, 2013). The host-specific microbial composition could be considered as part of the “parental effects” that were suggested to prepare offspring for an unpredictable environment (Cameron et al., 2008; Badyaev and Uller, 2009). Careful experimental design, including cross-fostering and *in vitro*-fertilization studies, sided by longitudinal experimental evolution studies are necessary to understand how long transgenerational effects may persist and to establish brain-gut-microbe bidirectional communications as a part of a “soft-inheritance” paradigm (Sweatt, 2013).

### Harmonization of the hologenome theory and apparent lamarckism with the neo-darwinist perspective

“ALL THE ACQUISITIONS OR LOSSES WROUGHT BY NATURE ON INDIVIDUALS, THROUGH THE ENVIRONMENT IN WHICH THEIR RACE HAS LONG BEEN PLACED, […] ALL THESE ARE PRESERVED BY REPRODUCTION TO THE NEW INDIVIDUALS WHICH ARISE […]”

(Jean-Baptiste Lamarck, 1809)

This quote summarizes the second law of the Lamarckian theory of evolution, which tried to explain evolutionary change by inheritance of acquired traits during the lifetime of an individual. Recent experimental findings, such as those discussed in this review, as well as the general interest in epigenetic transmission of phenotypic variation have repeatedly urged authors to partly resurrect this theory or at least raise the question, to what extent these Lamarckian aspects contribute to adaptation in a complementary way to the Darwin and Wallace theory of evolution (or, more accurately, the modern synthesis of genetics and evolution), (e.g., Smythies et al., 2014; Szyf, 2014). However, we feel that this perspective on epigenetics is of little avail for two main reasons. First, non-genetic information is ultimately coded by genetic information, i.e., there are genes for epigenetic processes, such as genes coding for histone- or DNA-modifying enzymes. These chromosomal genes clearly underlie the common, accepted Mendelian inheritance patterns and will be subject to natural selection or neutral evolution (drift) just as any other gene. Second, while the ability to pass environmental information to the next generation represents another source of variability that natural selection can act on, it is unlikely that the outcome of an epigenetic inheritance is stabilized and fixed through evolution. Together, these arguments imply that it will rather be the *possibility* to epigenetically inherit environmental information itself, i.e. the genes that encode the specific epigenetic machinery, that is positively selected for to promote flexibility. We have thus argued here that epigenetic mechanisms should rather be viewed as the evolutionary answer to nature's inherent unpredictability rather than constituting an alternative mode of evolution. This form of meta-adaptation or adaptability, which Mattick has referred to as “evolution has learnt how to learn” (Mattick, 2009), can be defined as the ability of a genetic system to produce and maintain potentially adaptive epigenetic variation and regulation. This definition is derived as an analogy to the concept of evolvability, defined as “the ability of the genetic system to produce and maintain potentially adaptive genetic variants” (Hansen, 2006; Pigliucci, 2008)

Appreciation grows for the fact that animals live in a bacterial world (McFall-Ngai et al., 2013), and no longer can the animal be viewed as separate from the microbes it requires to subsist, reproduce, and evolve over time. One principle that highlights this theme is the hologenome concept, which combines the host's multicellular genome and associated microbiome into a self-contained unit of selection that does not obviate other units of selection (Booth, 2014; Rosenberg and Zilber-Rosenberg, 2014), but recognizes that portions of the genome and microbiome are inseparable for encoding a viable organism and thus subject to co-evolution in similar ways to genes co-evolving within the nuclear genome. Indeed, Fitzpatrick recently identified circumstances under which this premise is fulfilled by deriving a mathematical framework based on ordinary population genetics, which provides further support for inclusion of non-genetic inheritance (such as symbionts, cultural traits and other epigenetic traits) in evolutionary concepts (Fitzpatrick, 2014).

In order to harmonize the different concepts of host-microbe co-dependencies mentioned in this article, it may help to take a gene-centric point of view—and thus consider symbiont genomes as extended chromosomes of the participating genomes within a holobiont. From this point of view, genes promoting the symbiosis by modulating their mutualistic counterpart would be positively selected, if this in turn promotes the overall fitness of the holobiont. The eukaryotic proportion provides a self-sustaining vehicle for the hologenome, while the microbial genes contribute by supplementing available genetic information (e.g., metabolic pathways) but also by modulating development and evolution of the holobiont in response to environmental stimuli.

### Closing remarks

“NO SELF IS OF ITSELF ALONE. […] THE ‘I’ IS CHAINED TO ANCESTRY BY MANY FACTORS […]. THIS IS NOT MERE ALLEGORY, BUT AN ETERNAL MEMORY.”

(Erwin Schrödinger, 1918)

In this theoretical article, we have gathered arguments for an integrated view on brain development and evolution, symbiosis, and RNA biology, which together frame social behavior (Figure 2): Pro-social behavior relies on proper brain development, which requires precisely timed gene expression, orchestrated by epigenetic regulatory mechanisms, such as histone modifications as well as the ncRNAome and other plastic RNA-based mechanisms (Figure 3). Given a potential co-evolution of social behavior in mammals and their microbiomes, brain development is furthermore particularly vulnerable to microbial signals. Gilbert et al. suggested that *“we have outsourced certain developmental signals”* to our microbiota (Gilbert et al., 2010). However, we need to consider that eukaryotic evolution has probably never seen a period without the presence of microbes, so that these particular developmental signals have never been produced “in-house” but are a result of co-evolution of host and microbiota. Gilbert and colleagues thus have a play on Kaufmann's evolutionary comment in stating that *“Almost all development may be co-development”* (Gilbert et al., 2010). In fact, together with Lombardo's theory of “access to mutualistic endosymbionts” (Lombardo, 2008), this perspective on brain-gut-microbiota co-development may hold a missing link in the “expensive-tissue hypothesis” (Aiello and Wheeler, 1995), in that it would explain how a socially-transmitted diverse and thus highly efficient microbiota could compensate for decreasing gastrointestinal tract size during primate brain enlargement by increasing nutrient availability from a broader range of sources.

It is now important to determine our psychobiotic endosymbionts, i.e., symbionts with a beneficial effect on mental health and neurodevelopment (Dinan et al., 2013), which constitute a positive relationship during development and which may aid treatment in case of disease. Understanding our long-term relationship with these beneficial friends will also have important implications for future research to provide lifestyle recommendations such as diet, hygiene and behavior in certain critical periods in life, including pregnancy and early education.

We may not yet fully understand the epigenetic potential of our bacterial friends and what their social benefits are, nonetheless we are beginning to appreciate the extent to which host-microbe interactions drive brain evolution and development.

### Conflict of interest statement

The authors declare that the research was conducted in the absence of any commercial or financial relationships that could be construed as a potential conflict of interest.
